# A single-surgeon experience in reconstruction of femoro-acetabular offset and implant positioning in direct anterior approach and anterolateral MIS approach with a curved short stem

**DOI:** 10.1007/s00402-021-03977-y

**Published:** 2021-06-02

**Authors:** Matthias Luger, Rainer Hochgatterer, Matthias C. Klotz, Jakob Allerstorfer, Tobias Gotterbarm, Bernhard Schauer

**Affiliations:** Department of Orthopaedics and Traumatology, Kepler University Hospital Linz, Krankenhausstrasse 9, 4020 Linz, Austria

**Keywords:** Total hip arthroplasty, short stem, Minimally invasive, direct anterior approach, Anterolateral approach, Offset reconstruction

## Abstract

**Purpose:**

Minimally invasive surgery using short stems in total hip arthroplasty gained more popularity. The differences in change of hip offset and implant positioning in minimally invasive approaches are not fully known. Therefore, this study was conducted to evaluate the difference in reconstruction of hip offset and implant positioning in direct anterior approach (DAA) and minimally invasive anterolateral approach (AL MIS).

**Methods:**

A single surgeon series of 117 hips (117 patients; mean age 65.54 years ± 11.47; index surgery 2014–2018) were included and allocated into two groups: group A (DAA) with 70 hips and Group B (AL MIS) with 47 patients operated. In both groups the same type of cementless curved short hip stem and press fit cup was used.

**Results:**

Both groups showed an equal statistically significant increase of femoral (*p *< 0.001) and decrease of acetabular offset (*p* < 0.001). Between both groups no statistically significant difference in offset reconstruction, leg length difference or implant positioning could be found. Leg length increased in both groups significantly and leg length discrepancy showed no difference (group A: − 0.06 mm; group B: 1.11 mm; *p *< 0.001). A comparable number of cups were positioned outside the target zone regarding cup anteversion.

**Conclusion:**

The usage of a curved short stem shows an equal reconstruction of femoro-acetabular offset, leg length and implant positioning in both MIS approaches. The results of this study show comparable results to the existing literature regarding change of offset and restoration of leg length. Malposition of the acetabular component regarding anteversion poses a risk.

## Introduction

In recent years minimally, invasive approaches gained more popularity [1, 2]. Minimally invasive approaches in total hip arthroplasty (THA) include the direct anterior (DAA), the anterolateral and the posterior approach to the hip [1]. Minimally invasive approaches (MIS) show the advantage of less blood loss [3], less perioperative pain and rapid recovery [4–6]. However, MIS approaches show the disadvantage of less surgical exposure posing the risk of potential implant malpositioning [7–9]. Femoral short stems are used more frequently in minimally invasive THA partly because of facilitating soft-tissue sparing implantation [10]. The development of short stems aimed at various issues, such as bone preservation of the proximal femur, the reduction of stress shielding and mid-thigh pain incidence [10–12].

Commonly used straight stems show excellent long-time outcomes [13], but have the disadvantage of limited ability to restore the femoral offset (FO) due to their straight stem design [14]. With modern femoral short stems, the correct restoration of natural hip anatomy should be facilitated [14, 15]. Besides leg length (LL), FO influences the postoperative outcome, dislocation rate, wear and revision rate [14]. FO is part of the abductor moment arm [14]. Restoration of the native FO increases range of motion, abductor muscle function and decreases polyethylene wear [14, 16–20]. Several studies even suggest a beneficial effect of an increased FO on abductor muscle force and joint reaction [21–23]. Given these findings, reconstruction of hip offset (HO) and LL shows a high clinical relevance. Recent studies suggest a sufficient reconstruction with new short stem systems [15] with better control of reconstructing FO compared to straight stem systems [24]. The possibility of correct offset reconstruction depends on the offset options of short stem systems [15]. Certain short stem systems with limited offset options show a significant loss of HO and increased valgus position [25].

Besides implant specific aspects the impact of the chosen approach on offset reconstruction is limited in the current literature. Studies show a potentially superior HO reconstruction and LL restoration in DAA compared to posterior and lateral approaches [26, 27]. The offset reconstruction of short stem systems in anterolateral approach also shows a good potential for correct reconstruction of offset and hip anatomy in THA [15, 24].

The data on reconstruction of HO and differences in offset reconstruction between different minimally invasive approaches is limited in current literature. In addition, minimally invasive approaches may have the risk of implant malpositioning. Therefore, this study was conducted to compare the difference of reconstruction of femoro-acetabular offset, leg length and implant positioning with a curved short stem in direct anterior approach and minimally invasive anterolateral approach.

## Materials and methods

### Study cohort

In this retrospective study a single-surgeon experience in reconstruction of femoro-acetabular offset and implant positioning in MIS THA using a DAA or AL MIS approach was analysed. 153 hips in 136 patients with index surgery between 2014 and 2018 were eligible. 35 hips were excluded. 15 patients were operated on both sides. In these cases, the first implantation was included because of statistical reasons. 4 bilateral one-stage implantations have been excluded, 5 hips because of peri- or postoperative complications and 3 hips because of missing landmarks on postoperative X-ray. 9 patients were lost to follow up. Therefore 117 hips in 117 patients were included in this study. Dependent on the surgical approach, patients were assigned either to group A (direct anterior approach) or group B (minimally invasive supine anterolateral approach). Group A consisted of 70 hips and group B of 47 hips. The study was approved by the institutional review board (EK-No.: 1239/2019). All procedures performed in studies involving human participants were in accordance with the ethical standards of the institutional and/or national research committee and with the 1964 Helsinki declaration and its later amendments or comparable ethical standards.

### Surgical procedure and implants

The procedures were performed by a single fellowship trained surgeon. DAA was carried out in a supine position on a standard operating table as previously described [28, 29]. Minimally invasive anterolateral approach also was carried on a standard operating table in supine position [30]. Flouroscopy was neither used for DAA nor anterolateral approach. The standardized peri- and postoperative protocol was identical in both groups, including single-shot antibiotics (Cefuroxime 1.5 g i.v. perioperatively), weight-bearing as tolerated, Indometacin 75 mg daily for the prevention of heterotopic ossification for 4 days and 40 mg low-molecular weight heparin or Rivaroxaban 10 mg for 28 days postoperatively as prophylaxis for deep vein thrombosis.

A cementless, curved short stem was used in all patients (Fitmore^®^ stem, ZimmerBiomet, Warsaw, IN, USA). The titanium alloy stem (Ti Al6V4) has a porolock Ti-VPS coating in the proximal part to enhance bone ingrowth and is available in four different neck angle options (127°, 129°, 137°, 140°) [31]. A cementless titanium press-fit cup with or without screws (Allofit^®^/-S, ZimmerBiomet, Warsaw, IN, USA) was used in all patients. In both groups the aim was a secure press-fit fixation, restoration of an equal leg length, reconstruction of the preoperative hip offset, cup inclination between 30 and 50° and cup anteversion between 10 and 30° [8, 32]. Preoperative planning of the prosthesis size and position was performed on anterior–posterior radiographic pelvis templates in all cases.

### Radiographic evaluation

**Fig. 1:**
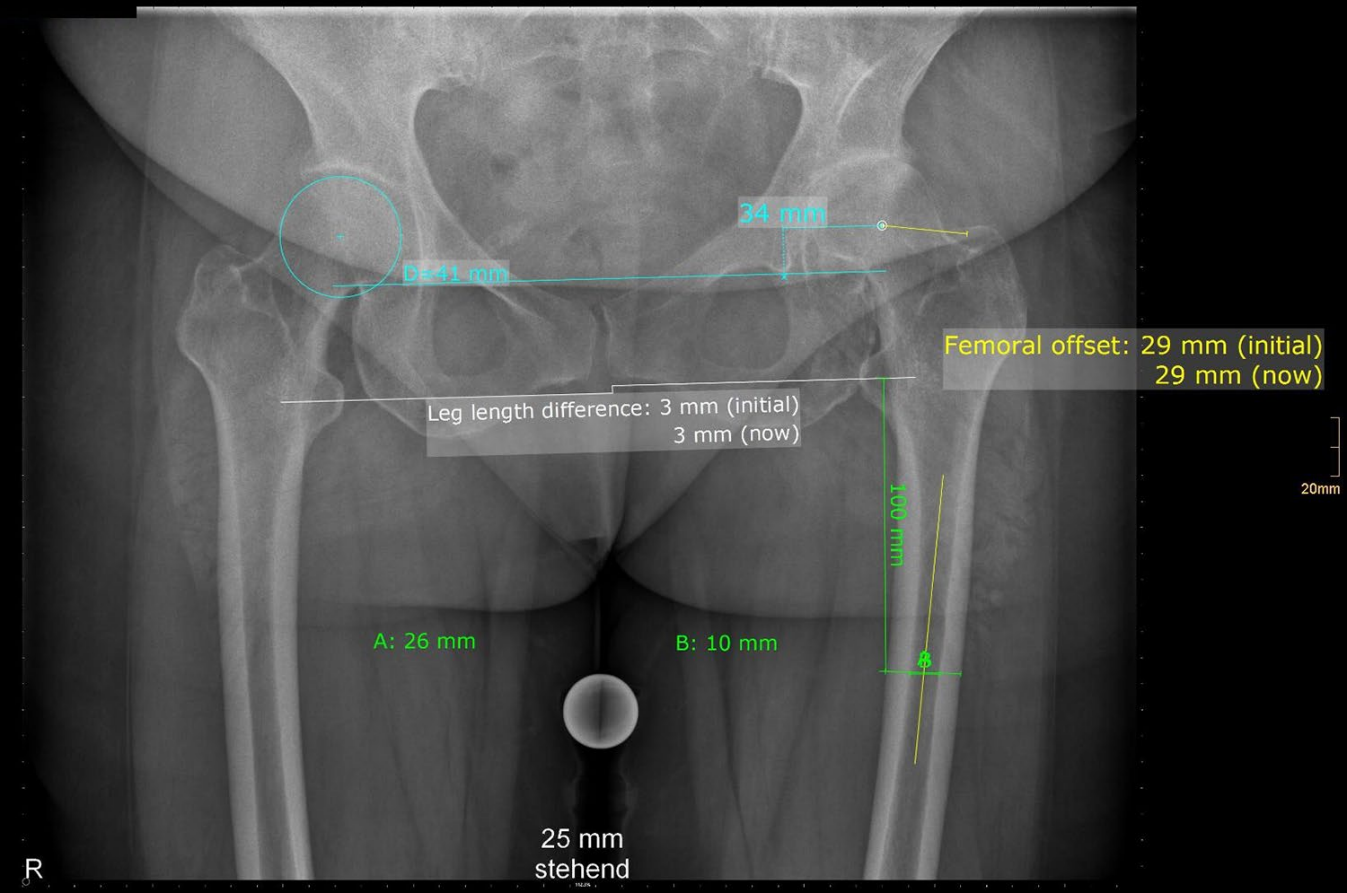
Preoperative X-ray with measurement of femoral offset (FO), acetabular offset (AO), leg length difference (LL) and Cortical Index (CI)

**Fig. 2:**
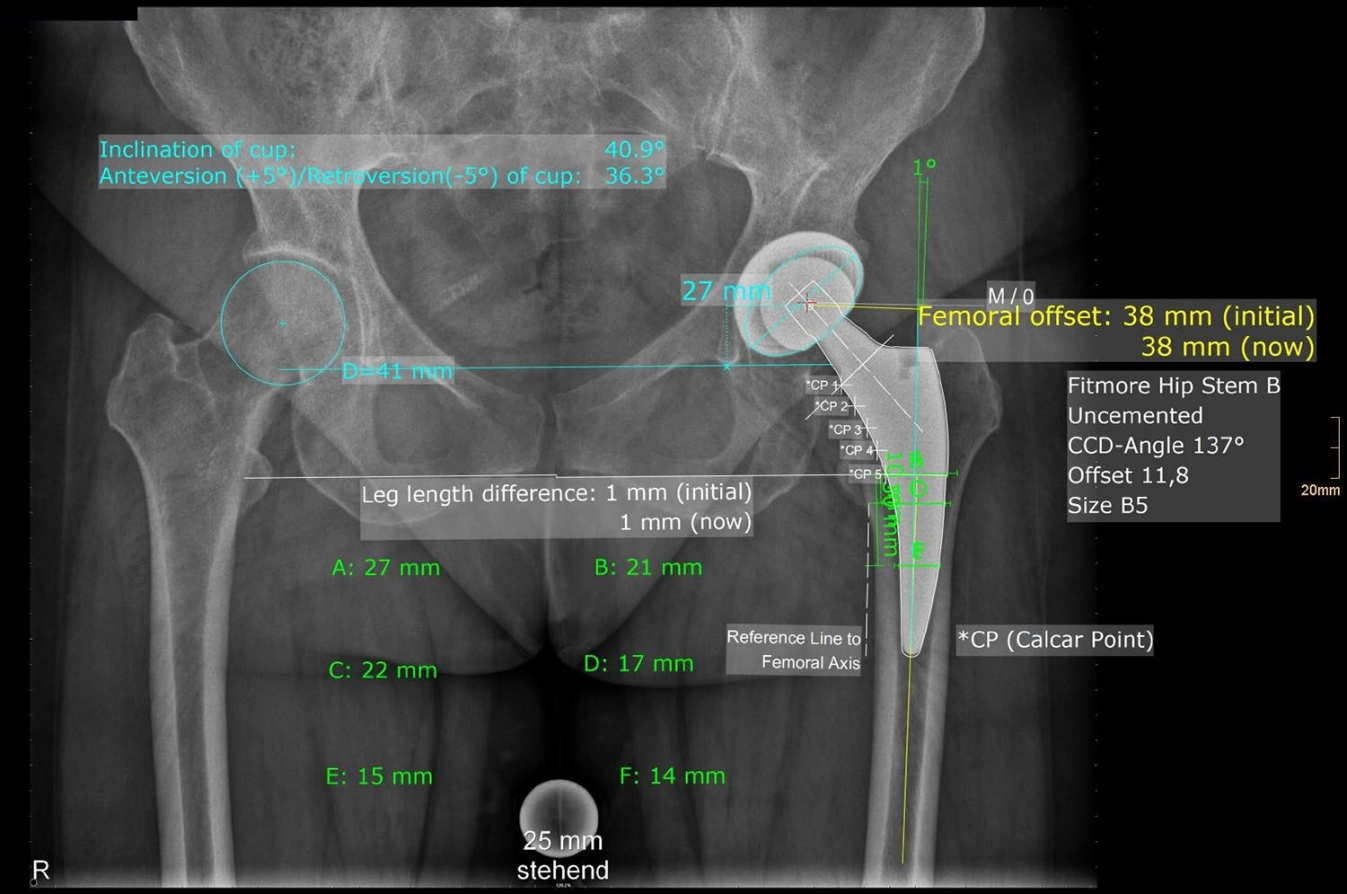
Postoperative X-ray with measurement with femoral offset (FO), acetabular offset (AO), leg length difference (LL), stem alignment, cup inclination and anteversion, Canal Fill Indices (CFI) I, II and III

Radiographic measurements were performed on pre and 3 month postoperative low centered anteroposterior (AP) radiographs of the pelvis. Radiographic measurement was performed on digital low-centered AP radiographs of the pelvis [33]. Radiographs were taken with the patient in standing position and with both legs in 15° internal rotation and the central beam was directed on the symphysis pubis [7]. To achieve an accurate measurement of the hip anatomy a double coordinate system was applied on both the preoperative and the postoperative images [15, 34]. Radiographic analysis was done using MediCAD^®^ Software V5.1 (HECTEC GmbH, Altdorf, Germany). The hip center of rotation (COR) was defined using a circle tool determining the diameter of the femoral head and its center [35]. The femoral offset (FO) was determined as the perpendicular distance between the COR and the proximal femoral shaft axis (FSA) [33, 35]. Acetabular offset (AO) was measured as the perpendicular distance between the COR and line T, with T being the perpendicular line on the transteardrop line (TT) through the ipsilateral teardrop figure [33]. Hip offset (HO) was calculated as the sum of FO and AO [33]. Stem alignment was measured as the difference in degrees between anatomic femoral shaft and vertical stem axis [36]. Cup inclination was defined as the angle between the TT line and the line connecting the most superior and inferior aspect of the cup. Cup anteversion was measured and calculated according to the formula by Lewinnek et al. [32], as recently validated by computer tomography based data [37]. Radiographic leg length (LL) was measured as the perpendicular distance between line TT and the middle of the lesser trochanter [7]. To characterize the anatomical shape of the proximal femur and the thickness of cortical bone, the cortical index (CI) according to Dorr et al. [38] were determined. A high CI indicates a thick cortical bone [38]. The canal fill index (CFI) was determined to evaluate the metaphyseal/diaphyseal filling of the femoral canal by the cementless stem implant on 3 different heights (CFI I: at the level of the LT, CFI II: 1 cm below the LT, CFI III: 3 cm below the LT). On each height, the horizontal diameter of the stem implant was measured and divided by the endosteal medullary canal diameter, multiplied by 100 [39, 40]. On preoperative X-ray FO, AO, HO and LL were measured. On postoperative X-ray FO, AO, HO, LL, cup inclination, cup anteversion, stem alignment, CI, CFI, CFII and CFIII were measured. Pre- and postoperative measurements are shown in Figs. 1 and 2.

### Statistical analysis

Statistical analysis was calculated with SPSS version 26 (IBM SPSS statistics, Chicago, IL, USA). Descriptive analysis was done for the parameters age, sex, offset and implant positioning. After exploratory data analysis, a Kolmogorov–Smirnov test was performed. As not all variables met the criteria for a normal distribution, non-parametric test was used. A Mann–Whitney *U* test was performed for testing between pre- and postoperative variables as well as between group A (DAA) and group B (AL MIS). The level of significance was *p *< 0.05.

## Results

A total of 117 hips in 117 patients have been included in this study. Gender showed a nearly equal distribution with 61 female and 56 male patients. The indication for THA was primary coxarthrosis in 105 patients, avascular necrosis in 7 patients and secondary arthrosis due to mild hip dysplasia (Crowe 1) in 5 patients. 70 hips (group A) were implanted through DAA and 47 hips (group B) were implanted through a minimally invasive anterolateral approach. Average age at operation in the general study cohort was 65.54 years (Min 30.79; Max 87.25; SD11.47). Average age at operation in group A was 65.86 years (Min 38.08; Max 86.91; SD 11.65) and in group B 65.05 years (Min 30.79; Max 87.25; SD 11.31).

### Offset reconstruction

FO increased in both groups significantly (*p *< 0.001) and AO decreased in both groups (*p *< 0.001). HO increased in both groups but only showed a statistical significance change for group A. The difference in pre- and postoperative FO, AO and HO showed no statistically significant difference in testing group A vs group B. LL also changed in both groups significantly (*p *< 0.001) and the LL difference showed no statistically significant difference in testing both groups. The detailed results for offset and leg length analysis are shown in Table 1.

**Table 1:** Results of offset analysis and leg length difference (all parameters in mm)

Variable	Group A (DAA)		Group B (AL MIS)	
	Preop	Postop	Preop	Postop
FO	40.83 ± 7.37	49.70 ± 8.45	42.38 ± 7.99	50.85 ± 8.66
*p* value	< 0.001		< 0.001	
FO difference	8.87 ± 5.94		8.47 ± 4.71	
*p* value	0.96			
AO	34.34 ± 4.56	29.47 ± 3.69	34.68 ± 4.41	29.15 ± 4.31
*p* value	< 0.001		< 0.001	
AO difference	− 4.87 ± 3.83		− 5.53 ± 4.33	
*p* value	0.577			
HO	75.17 ± 9.42	79.17 ± 10.3	77.06 ± 9.81	80 ± 10.07
*p* value	0.018		0.165	
HO difference	4 ± 4.98		2.94 ± 4.41	
*p* value	0.435			
LL	− 3.57 ± 5.49	− 0.06 ± 4.49	− 3.66 ± 6.02	1.11 ± 5.13
*p* value	< 0.001		< 0.001	
LL difference	3.51 ± 5.42		4.77 ± 4.66	
*p* value	0.179			

### Implant positioning

Implant positioning showed in both groups similar results for inclination and anteversion. The acetabular components showed an inclination within the defined target zone in 92.9% of cases for group A and 83% for group B. A statistically significant difference for cups without the target zone could not be found between both groups. Testing for cups outside the target zone regarding anteversion also showed no statistically significant difference. Both groups showed a similar number of cups outside the target zone regarding anteversion with 42.9% of cups in group A and 46.8% of cups in group B. Stem alignment showed an average varus angle of 5.33° in group A and 5.46° in group B without any statistical significance. In addition, the evaluation of canal filling index showed no statistically significant difference for CI, CFI, CFII and CFIII between both groups. Detailed results of implant positioning are shown in Table 2.

**Table 2:** Results of implant positioning

Variable	Group A (*n* = 70)	Group B (*n* = 47)
Cup inclination (°)	40.63 ± 5.95	43.84 ± 6.25
Within target zone	65 (92.9%)	39 (83%)
Outside target zone	5 (7.1%)	8 (17%)
*p* value	0.097	
Cup anteversion (°)	28.62 ± 4.59	30.82 ± 6.08
Within target zone	40 (57.1%)	25 (53.2%)
Outside target zone	30 (42.9%)	22 (46.8%)
*p* value	0.675	
Stem alignment (°) (varus/valgus)	5.33 ± 3.54	5.46 ± 3.44
*p* value	0.833	
CI (%)	59.72 ± 5.62	59.70 ± 4.56
*p* value	0.761	
CFI (%)	79.8 ± 7.26	80.25 ± 8.29
*p* value	0.717	
CFII (%)	82.7 ± 7.6	83.07 ± 7.9
*p* value	0.703	
CFIII (%)	85.85 ± 10.17	83.81 ± 10.72
*p* value	0.427	

## Discussion

Short stems were introduced partly because of theoretical advantage of better offset reconstruction [15]. A better reconstruction of femoro-acetabular offset and the natural anatomy of the hip is associated with better clinical and functional outcome [21–23]. The results show no statistically significant difference in comparing both minimally invasive approaches. Both approaches result in similar reconstruction of femoro-acetabular offset and leg length. HO increases in both groups but only shows a significant increase in DAA. However, the difference in increase in HO is 4 mm in DAA and 2.94 for AL MIS. The smaller group size in Group B (AL MIS) could be a factor of a missing statistical significance of pre- and postoperative HO. Furthermore, in testing between HO difference in DAA vs AL MIS no significant difference could be found.

Kutzner et al. [15] showed a statistically significant increase of FO and HO as well as a statistically significant decrease of AO in 109 patients in THA with a femoral neck preserving short stem (Optimys^®^ Mathys Ltd, Bettlach, Switzerland) in minimally invasive supine anterolateral approach. The average increase of FO was 5.8 mm and 2.1 mm for HO. An average reduction of 3.7 mm was found in AO. Similar findings have been published by Erivan et al. [24] for the same short stem system. Erivan et al. [24] found a statistically significant increase of 4.7 mm in FO in 100 patients with THA with the same short stem system in anterolateral approach compared to 7.2 mm in straight stem arthroplasty in a matched control group with 100 patients. Compared to a straight system a statistically significant difference in increasing FO could be found with *p *= 0.0152 [24]. Therefore, the short stem system was considered to provide better control in restoration of femoral offset [24]. Similar to these findings FO increased in THA with the femoral neck sacrificing curved short stem (Fitmore^®^, ZimmerBiomet) in this study. While offset reconstruction in femoral neck sparing short stems is controlled by defining the correct femoral neck osteotomy, Fitmore short stem provides offset reconstruction by 4 different offset options. With this short stem system an increase of FO with 8.87 mm could be found in DAA and 8.47 mm in AL MIS approach. The findings of this study show a slightly higher increase in FO in both minimally invasive approaches compared to similar studies [15, 24]. A reason for higher increase in FO may be a result of a higher decrease of AO and medialization of the acetabular component. AO decreased with 4.87 mm in DAA and 5.53 mm in AL MIS compared to 3.7 mm in a comparable study [15]. A decrease of AO is typical in cementless press-fit acetabular cups. The medialization of the center of rotation is therefore compensated in increasing FO to achieve a stable hip joint and to prevent limitation in functional outcome because of a decreased HO. This is supported by the findings for both groups in this study. The results for increase of HO are comparable for both groups with 4 mm for DAA and 2.94 mm for AL MIS compared to 2.1 mm in a comparable study [15].

Leg length and leg length difference is also an important factor for a good clinical outcome and patient satisfaction. This study showed a statistically significant (*P *< 0.001) increase of LL in both groups with an average postoperative LL discrepancy of − 0.06 mm for DAA and 1.11 mm in AL MIS. These findings show a comparable restoration of LL for both approaches with Fitmore short stem. The findings are also comparable to findings in other studies with an increase of LL of 2.4 mm and 2.86 mm [15, 24] and average postoperative LL difference of 1.17 mm [15]. However, the comparison of LL discrepancies show limitations because of different measuring techniques. In this study a radiological measurement of LL and LL discrepancy was used, while other studies use clinical measurement. Innmann et al. [40] compared reconstruction of individual hip anatomy in patients with a native contralateral hip. An average postoperative LL discrepancy of − 3 mm could be found in the group with Fitmore short stem.

Implant positioning showed no statistically significant difference for anteversion, inclination and stem alignment. Studies suggest the risk of varus malpositioning of femoral straight shafts in DAA [41]. The findings in this study shows no difference in stem alignment with Fitmore short stem for DAA and AL MIS approach, but shows a tendency of a varus implantation with an average varus degree of 5.33° for DAA and 5.46° for AL MIS approach. This could be a result of the generally calcar guided implantation technique of this stem system. MIS approaches (anterolateral, two-incision, lateral and posterior) were described as a risk factor for cup malpositioning in large patient series [8, 9]. Innmann et al. [7] found a statistically significant malpositioning of acetabular components in minimally invasive anterolateral approach according to Röttinger [4] compared with transgluteal approach. A positioning within the target zone was demonstrated in 89% for inclination and 71% for anteversion in minimally invasive anterolateral approach. The findings in this study show similar results for inclination within the target zone of 92.9% in DAA and 83% in minimally invasive supine anterolateral approach. Regarding anteversion this study suggests a lower number of cups within the target zone with only 57.1% and 53.2% for DAA and AL MIS. Soderquist et al. [42] described a comparable rate of 85% within the same target zone for inclination and 61% for anteversion in freehand placement of acetabular cup in DAA. Rathod et al. [43] reported a higher number of cups within the target zone for DAA with fluoroscopy. However, DAA with fluoroscopy was compared to posterior approach without fluoroscopy. Bingham et al. [44] compared cup placement in DAA with and without fluoroscopy and did not find any significant difference in cup positioning and leg length difference. We postulate, that the risk for cup malpositioning is comparable in DAA and MIS anterolateral approach, because of the reduced surgical exposure in both approaches.

Regarding metaphyseal/diaphyseal filling the canal fill index was evaluated in this study. Innmann et al. [40] found an average CFI of 85.6%, CFII of 90.4% and CFIII of 85.2% for Fitmore short stem in a modified lateral approach according to Bauer and Russe [45]. The findings in this study show slightly lower canal fill indicis for both approaches. Both minimally invasive approaches show slightly lower results with an average CFI of 79.8% and 80.25%, an average CFII of 82.7% and 83.07% an average CFIII of 85.85% in DAA and 83.81% AL MIS approach. These findings for minimally invasive approaches was also found in comparable studies with canal fill indices ranging from 77 to 94% in DAA with Fitmore short stem [46]. These lower canal fill indices could be a result of slightly impaired femoral broaching due to reduced femoral exposure in minimally invasive approaches.

Limitations of this study are the retrospective study design and the low case number. The retrospective design could lead to a selection bias because of missing prospective randomization. An additional limitation is the evaluation on plain two-dimensional digital radiographs. These can result in a bias because of poor quality of X-ray, e.g., in missing landmarks. This was address with exclusion of patients with insufficient X-rays for radiographic evaluation of the chosen parameters. The use of standardized radiographic techniques and previously described and used measurement techniques for evaluating offset and implant parameters. The strengths of this study first result of being a single-surgeon experience. Second, the measurements are easily reproducible and comparable to existing data.

## Conclusion

Fitmore curved short stem shows an equal reconstruction of femoro-acetabular offset, leg length and implant positioning in direct anterior approach and minimally invasive supine anterolateral approach. The results of this study show comparable results with Fitmore stem to the existing literature regarding offset reconstruction and restoration of leg length. Malposition of the acetabular component regarding anteversion poses a risk and surgeons should be aware when using minimally invasive approaches. Regarding reconstruction of femoro-acetabular offset, leg length and implant positioning direct anterior and minimally invasive supine anterolateral approach show sufficient results.
